# Complete plastome sequence of *Erythropalum scandens* (Erythropalaceae), an edible and medicinally important liana in China

**DOI:** 10.1080/23802359.2017.1413435

**Published:** 2018-02-01

**Authors:** Zhi-Xin Zhu, Jian-Hua Wang, Ya-Cheng Cai, Kun-Kun Zhao, Michael J. Moore, Hua-Feng Wang

**Affiliations:** a Hainan Key Laboratory for Sustainable Utilization of Tropical Bioresources, Institute of Tropical Agriculture and Forestry, Hainan University, Haikou, China;; b State Key Laboratory of Biocontrol and Guangdong Provincial Key Laboratory of Plant Resources, School of Life Sciences, Sun Yat-sen University, Guangzhou, China;; c Department of Biology, Oberlin College, Oberlin, OH, USA

**Keywords:** Erythropalaceae, *Erythropalum scandens*, Illumina sequencing, Plastome, Phylogenetic analysis, Santalales

## Abstract

*Erythropalum scandens* (Erythropalaceae, Santalales) is a large liana distributed in alluvial and riparian forests of Southeast Asia. Here we report and characterize the complete plastid genome sequence of *E. scandens* in an effort to provide genomic resources useful for developing its medicinal and edible value. The complete plastome is 156,154 bp in length and contains the typical structure and gene content of angiosperm plastomes, including two Inverted Repeat (IR) regions of 26,394 bp, a large single-copy (LSC) region of 84,799 bp and a small single-copy (SSC) region of 18,567 bp. The plastome contains 112 genes, consisting of 79 unique protein-coding genes, 29 unique tRNA genes and four unique rRNA genes. The overall A/T content in the plastome of *E. scandens* is 62.01%. Phylogenetic analyses were performed using the entire plastome, including genes, spacers and introns, which recovered *E. scandens* as sister to remaining Santalales with complete plastome sequences.

*Erythropalum scandens* Blume (Erythropalaceae sensu Nickrent et al. 2010) is a large liana endemic to the forests of Hainan Province, China (Qiu and Gilbert 2003). It is used as an edible wild vegetable in tropical and subtropical areas of southeast Asia, e.g. local peoples from Guangxi, Guangdong, Hainan and southern Yunnan provinces in China have traditionally collected and eaten its young leaves and stems. It contains resins, phenolic and triterpene medicinal ingredients in the roots. The main components of the resin include sumaresinolic acid and coniferyl cinnamate (Qiu and Gilbert 2003). Consequently, genetic and genomic information is urgently needed to promote the medicinal and edible value of *E. scandens*. Here, we report and characterize the complete plastome of *E. scandens* (GenBank accession number: this study) based on Illumina paired-end sequencing data (Illumina, San Diego, CA). This is the first report of a complete plastome for the genus *Erythropalum* and Erythropalaceae, which occupies an important phylogenetic position near the base of the large angiosperm order Santalales (Nickrent et al. 2010).

Leaf material of *E. scandens* was sampled from Diaoluo Mountain National Nature Reserve in Hainan province of China (109.88°E, 18.67°N). A voucher specimen (Wang et al. B40) was deposited in the herbarium of the Institute of Tropical Agriculture and Forestry, Hainan University, Haikou, China. The modified CTAB method of Doyle and Doyle (1987) was used to extract total genomic DNA from leaves quickly frozen with dry ice. 1 μg of genomic DNA was used for Illumina library preparation, using version 3 chemistry. Paired-end, 150 bp reads were sequenced using an Illumina HiSeq 2500 platform at the Guangzhou Novel-seq Biotechnology Co, Ltd (Guangzhou, China). Reads were trimmed and those with >10% Ns or with >10% low-quality (Q ≤ 5) bases were filtered out using NGSQC-Toolkit v2.3.3 (Patel and Jain 2012). Cleaned reads were assembled against the plastome of *Stephania japonica* (NC_029432_1) (Nock et al. 2014) using MITO bim v1.8 (Hahn et al. 2013).

The plastome was annotated using Geneious R8.0.2 (Biomatters Ltd., Auckland, New Zealand) against the plastome of *Stephania japonica*. The annotation was corrected with DOGMA (Wyman et al. 2004). A circular plastome map was generated using OGDRAW (http://ogdraw.mpimp-golm.mpg.de/) (Lohse et al. 2013).

The plastome of *E. scandens* was found to possess a total length 156,154 bp with the typical quadripartite structure of angiosperms, containing two Inverted Repeats (IRs) of 26,394 bp separated by a large single-copy (LSC) region and a small single-copy (SSC) region of 84,799 and 18,567 bp, respectively. The plastome was found to contain 125 genes, including 79 protein-coding genes (six of which are duplicated in the IR), four ribosomal RNA genes, and 29 tRNA genes (seven of which are duplicated in the IR). Among these genes, 14 genes (*trn*A-UGC*, trn*I-GAU*, trn*K-UUU*, trn*L-UAA*, trn*V-UAC*, atp*F*, ndh*A*, ndh*B*, pet*B*, pet*D*, rpo*C1*, rpl*2*, rpl*16*, rps*16) possessed a single intron and three genes (*ycf3, clp*P*, rps12*) had two introns. The gene *rps12* was found to be trans-spliced, as is typical of angiosperms. The overall A/T content of the plastome was 62.01%, while the corresponding values of the LSC, SSC and IR regions were 63.77%, 67.66% and 57.18%, respectively.

We used RAxML (Stamatakis 2006) with 1000 bootstraps under the GTRGAMMAI substitution model to reconstruct a maximum-likelihood (ML) phylogeny of 11 published complete plastomes of Santalales and Caryophyllales, using *Hydrangea luteovenosa* (Hydrangeaceae, Cornales) as an outgroup. The phylogenetic analysis recovered *E. scandens* as sister to remaining Santalales, with maximum bootstrap support (Figure 1). The plastome reported here will provide a useful resource for development of the medicinal and edible value of *E. scandens,* as well as for phylogenetic studies of Santalales.

**Figure 1.**
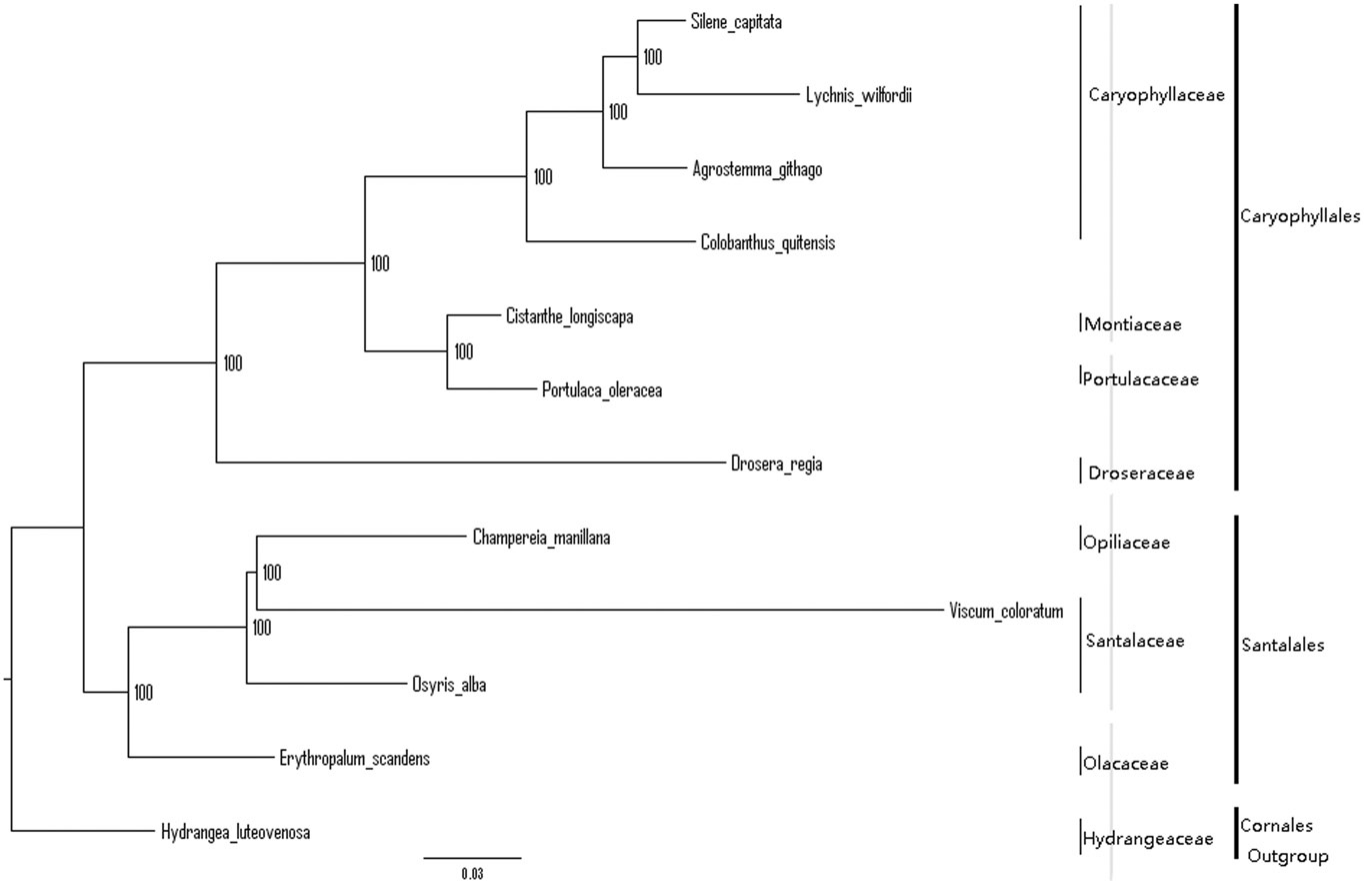
The best ML phylogeny recovered from plastomes included in this study. Accession numbers: *Erythropalum scandens* (This study)*, Osyris alba* NC_027960.1*, Viscum coloratum* NC_035414.1, *Champereia manillana* NC_034931.1, *Agrostemma githago* NC_023357.1, *Colobanthus quitensis* NC_028080.1, *Lychnis wilfordii* NC_035225.1, *Silene capitata* NC_035226.1, *Drosera regia* NC_035415.1, *Cistanthe longiscapa* NC_035140.1, *Portulaca oleracea* NC_036236.1, *Hydrangea luteovenosa* NC_035662.1 (lower in the figure).
